# The long-term outcomes of early abdominal wall reconstruction by bilateral anterior rectus abdominis sheath turnover flap method in critically ill patients requiring open abdomen

**DOI:** 10.1186/s13017-018-0200-7

**Published:** 2018-09-04

**Authors:** Masatoku Arai, Shiei Kim, Hiromoto Ishii, Jun Hagiwara, Shigeki Kushimoto, Hiroyuki Yokota

**Affiliations:** 1 Department of Emergency and Critical Care Medicine, Nippon Medical School, 1-1-5 Sendagi, Bunkyo-ku, Tokyo, 113-8603 Japan; 2 Division of Emergency and Critical Care Medicine, Tohoku University Graduate School of Medicine, 1-1 Seiryo-machi, Aoba-ku, Sendai, Miyagi 980-8574 Japan

**Keywords:** Open abdomen, Negative pressure wound therapy, Abdominal wall reconstruction, Abdominal compartment syndrome

## Abstract

**Background:**

In a previous study, we reported the usefulness of early abdominal wall reconstruction using bilateral anterior rectus abdominis sheath turnover flap method (turnover flap method) in open abdomen (OA) patients in whom early primary fascial closure was difficult to achieve. However, the long-term outcomes have not been elucidated. In the present study, we aimed to evaluate the procedure, particularly in terms of ventral hernia, pain, and daily activities.

**Methods:**

Between 2001 and 2013, 15 consecutive patients requiring OA after emergency laparotomy and in whom turnover flap method was applied were retrospectively identified. The long-term outcomes were evaluated based on medical records, physical examinations, CT imaging, and a ventral hernia pain questionnaire (VHPQ).

**Results:**

The turnover flap method was applied in 2 trauma and 13 non-trauma patients.

In most of cases, primary fascial closure could not be achieved due to massive visceral edema. The turnover flap method was performed for abdominal wall reconstruction at the end of OA. The median duration of OA was 6 (range 1–42) days. One of the 15 patients died of multiple organ failure during initial hospitalization after the performance of the turnover flap method. Fourteen patients survived, and although wound infection was observed in 3 patients, none showed enteric fistula, abdominal abscess, graft infection, or ventral hernia during hospitalization. However, it was found that 1 patient developed ventral hernia during follow-up at an outpatient visit. Nine of 14 patients were alive and able to be evaluated with a VHPQ (follow-up period: median 10 years; range 3–15 years). Seven out of nine patients were satisfied with this procedure, and none complained of pain or were limited in their daily activities.

**Conclusions:**

Based on the results of this study, early abdominal reconstruction using the turnover flap method can be considered to be safe and effective as an alternative technique for OA patients in whom primary fascial closure is considered difficult to achieve.

## Background

Open abdomen (OA) in critically ill patients is reported to be necessary for saving lives [1]. Abdominal compartment syndrome, damage control surgery, and abdominal sepsis are the indications for OA [1, 2]. However, it is also known that a prolonged duration of OA may result in severe complications, including the development of an abscess, enteric fistula, graft infection, and extensive ventral hernia [1, 2]. It has recently been recommended that in all patients with an OA, every effort should be exerted to achieve primary fascial closure as soon as the patient can physiologically tolerate it [3, 4].

Temporary abdominal closure (TAC) is an important procedure for accomplishing primary fascial closure. Although many different TAC techniques have evolved [1, 2, 4], there are some cases of prolonged TAC in which primary fascial closure is difficult to achieve [1, 2, 4]. Thus, early abdominal wall reconstruction may be proposed to avoid severe complications in acute phase of OA. Component separation (CS) technique, a tissue transfer technique that has mostly been used in elective abdominal wall reconstruction for the repair of ventral hernias, was recently reported to show favorable results in early abdominal wall reconstruction [5]. Bilateral anterior rectus abdominis sheath turnover flap method (turnover flap method), which has been reported to be useful as an alternative tissue transfer technique, may be also considered as a technique for early abdominal wall reconstruction [6]. However, the long-term outcomes of this method have not been elucidated. The aim of the present study was to evaluate the long-term outcomes of early abdominal wall reconstruction by the turnover flap method in critically ill patients who required OA.

## Methods

This study received approval from the Institutional Review Board of Nippon Medical School Hospital. The study population included all patients who were admitted to the Department of Emergency and Critical Care Medicine of Nippon Medical School between January 2001 and December 2013 and who underwent a procedure using the turnover flap method. The patients using this procedure were retrospectively identified from their medical records. The turnover flap method was performed at the end of OA for early definitive reconstruction of the abdominal wall for OA patients in whom early primary fascial closure was difficult to achieve. A total of 15 consecutive patients (trauma, *n* = 2; non-trauma patients, *n* = 13) were considered suitable for inclusion during the specified study period. All medical records from the initial hospitalization at the time OA was created to the time of follow-up were reviewed for this study to obtain information, including demographics, mechanisms, indications and duration of OA, types of TAC, postoperative complications, and outcomes. Ventral hernia was defined as a fascial defect at the site of the midline incision with or without protrusion of intra-abdominal contents, detected on a physical examination or CT. Bulging without a fascial defect detected on a physical examination or CT was registered, but not defined as a ventral hernia. The data were expressed as the mean ± standard deviation (SD).

### Ventral hernia pain questionnaire (VHPQ)

The questionnaire consists of 20 questions and takes approximately 5 min to complete. It concerns the level and duration of pain, the impact on daily activities, patient satisfaction, and how physically demanding the patients regarded their occupation [7].

### Temporary abdominal closure

In the present study, Bogota bag alone was used for 6 patients, negative pressure wound therapy (NPWT) alone was used for 4 patients, Bogota bag was changed to NPWT for 2 patients, and combination of NPWT and mesh-mediated traction [8, 9] was used for 3 patients. These dressings are changed every 2–3 days in the operating room or at the bedside. If the abdominal fascia could be fully approximated without tension during a dressings change, then primary fascial closure was performed. If it was not possible to accomplish definitive fascial closure with the use of TAC alone, then the turnover flap method was applied in the early phase of abdominal wall reconstruction.

### The procedure for the turnover flap method

**Fig. 1:**
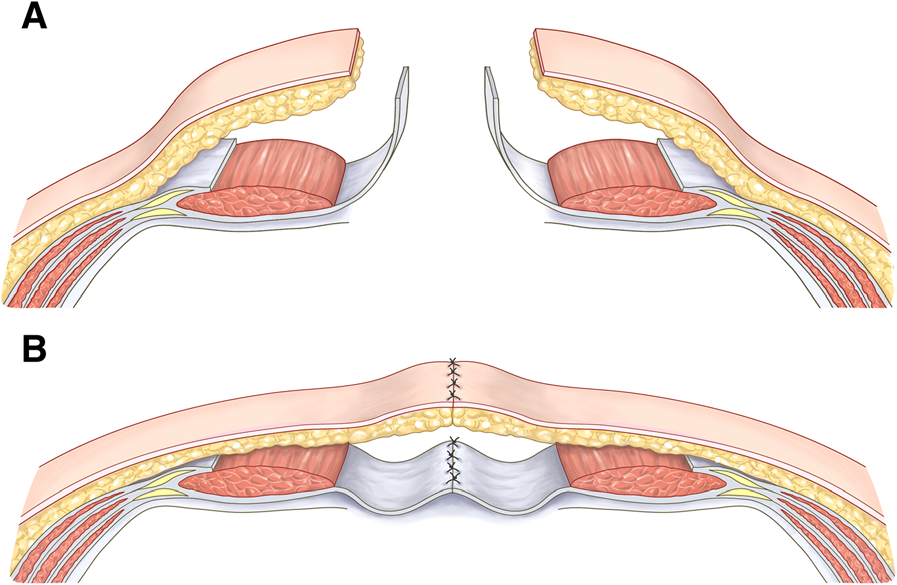
Schematic illustration of the technique for bilateral anterior rectus abdominis sheath turnover flap method. **a** First, the skin and subcutaneous tissue are separated from the anterior rectus sheath as a flap bilaterally beyond the lateral border of the rectus abdominis sheath. A longitudinal incision is then made in each anterior rectus sheath ≥ 1 cm inside the lateral border. After confirming the presence of the rectus abdominis muscle, the incision is extended the entire length of the anterior rectus sheath. The anterior rectus sheath is then dissected from the lateral to medial face, freeing it from the rectus abdominis muscles. **b** It is reflected medially and approximated with interrupted absorbable sutures to cover the abdominal contents. The skin is closed primarily or secondarily

We reported the turnover flap method in 2007 [6]. First, the skin and subcutaneous tissue are separated from the anterior rectus sheath as a flap bilaterally beyond the lateral border of the rectus abdominis sheath (Fig. 1a). A longitudinal incision is then made in each anterior rectus sheath ≥ 1 cm inside the lateral border. After confirming the presence of the rectus abdominis muscle, the incision is extended the entire length of the anterior rectus sheath (Fig. 1a). The anterior rectus sheath is then dissected from the lateral to medial face, freeing it from the rectus abdominis muscles (Fig. 1a). It is then reflected medially and approximated with interrupted absorbable sutures to cover the abdominal contents. The skin is closed primarily or secondarily (Fig. 1b).

Points to note are as follows: The longitudinal incision of the anterior rectus sheath should be started at the upper or lower surface, as the largest abdominal wall gap is mostly in the mid-abdomen, where a wide flap is needed to approximate the fascia. The linea alba should be kept intact to serve as a medial hinge. If the median incision is separated from the linea alba in the first laparotomy, absorbable suture repair must be performed. If the anterior rectus sheath is injured during this procedure, it can be repaired using absorbable sutures (dissecting the anterior rectus sheath from the intersectiones tendineae can be sometimes difficult due to firm adhesion).

The skin along the abdominal wall newly reconstructed using turnover flaps is sometimes too tight for primary closure; in such cases, a bipedicled skin flap is considered effective [10].

## Results

### Patient characteristics

The turnover flap method was performed in the treatment of 2 trauma patients and 13 non-trauma patients. The median patient age was 70 (range 8–90) years. Eleven patients were men, and the other 4 were women. One of the most common indications for OA was the prevention of ACS in cases that primary fascial closure was difficult to achieve (12/15), in which aggressive resuscitation was ongoing for profound shock due to severe hemorrhage or sepsis and visceral edema had the potential to increase after the initial operation. The median duration of OA was 6 (range 1–42) days (Table 1).

**Table 1:** Characteristics in patients undergoing turnover flap method

Patients	15	
Age (median, range)	70	8–90
Sex ratio (male)	11	73.3%
Diagnosis		
Non-trauma	13	86.7%
RAAA	8	53.3%
Diffuse peritonitis	3	20.0%
Severe acute pancreatitis	1	6.7%
Hemorrhagic gastric ulcer	1	6.7%
Trauma	2	13.3%
Hepatic injury, SMV injury, and pelvic fracture	1	6.7%
Diaphragmatic and duodenal injury and pelvic fracture	1	6.7%
Indication of OA		
Prevention of ACS	12	80.0%
ACS	1	6.7%
Damage control surgery	2	13.3%
Duration of OA (day: median, range)	6	1–42

*ACS* abdominal compartment syndrome, *OA* open abdomen, *RAAA* ruptured abdominal aortic aneurysm, *SMV* superior mesenteric vein

### Complications during hospitalization

Wound infection was observed in 3 patients. In one patient with major wound infection, the midline skin closure showed dehiscence; however, since the approximated fascial flap appeared to be intact, the skin was closed secondarily. No other wound complications (e.g., hematoma, seroma, or skin necrosis) were detected. One patient with a ruptured abdominal aortic aneurysm died of multiple organ failure (MOF) after the performance of the turnover flap method during hospitalization. None of the 14 patients surviving hospitalization developed enteric fistula, abdominal abscess, graft infection, or ventral hernia.

### Long-term follow-up

Five out of 15 patients died in 2015. Nine patients were able to be evaluated based on physical examinations, CT imaging, and the VHPQ. Their median follow-up period after abdominal wall reconstruction using the turnover flap method was 10 (range 3–15) years (Table 2).

**Table 2:** Characteristics in the long-term follow-up of patients undergoing turnover flap method

Case	Gender	Diagnosis	Reason for OA	TAC	Duration of OA (days)	Complications	Length of hospital stay (days)	Follow-up period (years)
Non-trauma								
1	Male	Ruptured AAA	Prevention of ACS	Silo	6	Ventral hernia	74	12
2	Male	Severe acute pancreatitis	ACS (IAP 30 mmHg)	Silo, NPWT	31	None	120	13
3	Male	Ruptured AAA	Prevention of ACS	NPWT	8	None	36	10
4	Female	Ruptured AAA	Prevention of ACS	NPWT	3	None	60	6
5	Male	Ruptured AAA	Prevention of ACS	Mesh traction + NPWT	8	None	60	3
6	Male	Diffuse peritonitis	Abdominal sepsis	Mesh traction + NPWT	42	None	56	4
7	Female	Diffuse peritonitis	Abdominal sepsis	Mesh traction + NPWT	31	None	68	3
Trauma								
1	Male	Hepatic and SMV injury, pelvic fracture	Damage control	Silo	6	None	101	15
2	Female	Diaphragmatic and duodenal injury, pelvic fracture	Damage control	Silo, NPWT	30	None	105	12

*ACS* abdominal compartment syndrome, *OA* open abdomen, *AAA* abdominal aortic aneurysm, *SMV* superior mesenteric vein

**Fig. 2:**
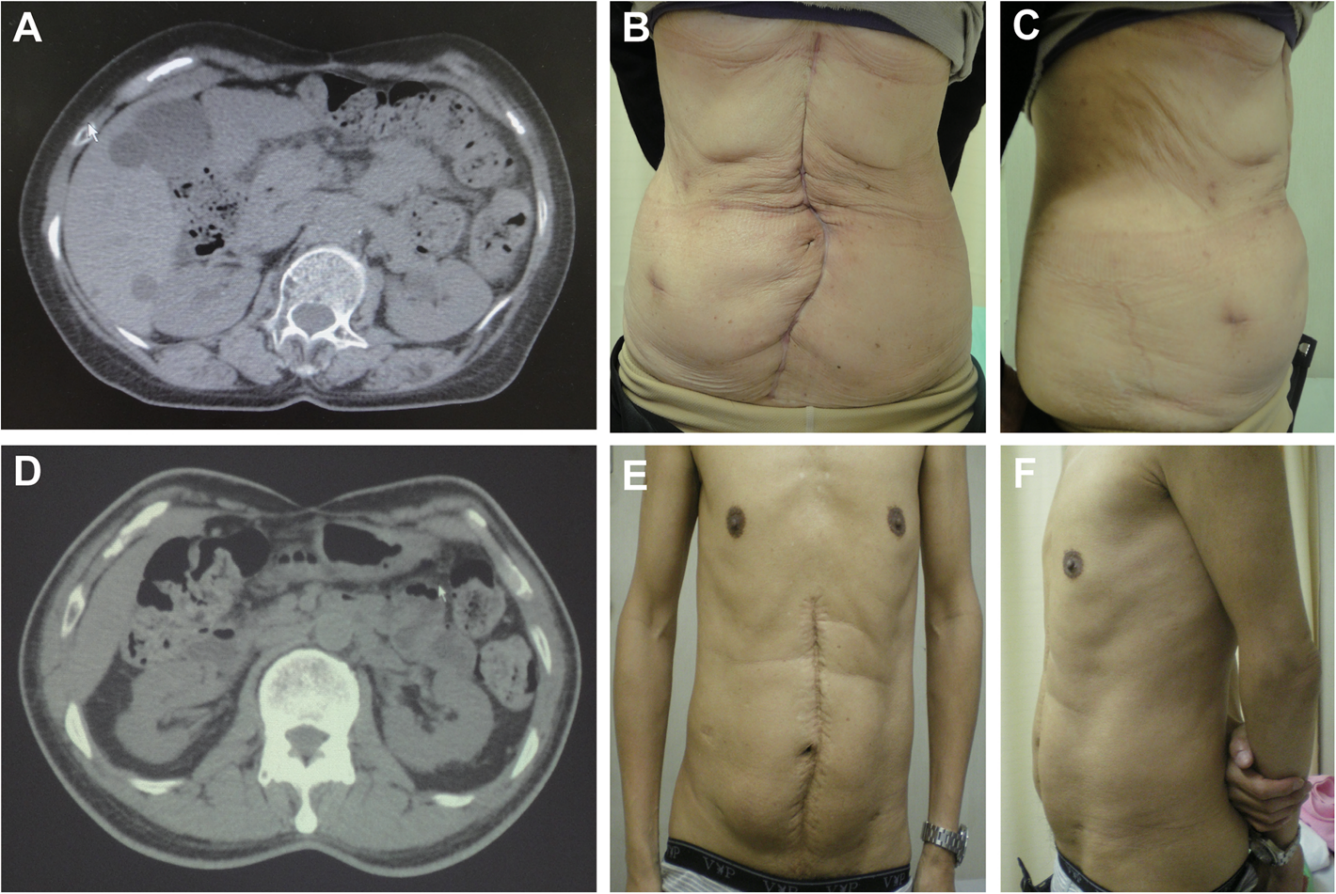
The CT findings and views of the abdominal wall. The upper panels show images obtained 6 years after performing the turnover flap method (**a**, **b**, **c**). The lower panels show images obtained 10 years after turnover flap method (**d**, **e**, **f**). The CT scans show that the appearance of flaps created using bilateral anterior rectus abdominis sheath looks similar to after onlay mesh repair (**a**, **d**). Anteroposterior views (**b**, **e**) and lateral views (**c**, **f**) of the abdominal wall in the standing position. Although slight lower abdominal bulging can be observed in the lateral views of both patients, no abdominal hernia is evident

In CT imaging of cases in which the turnover flap method was applied, the flaps using the bilateral anterior rectus abdominis sheath looked similar to after onlay mesh repair (Fig. 2a, d). In a view of the abdominal wall, although slight lower abdominal bulging can be observed in lateral views, ventral hernia was not evident (Fig. 2b, c, e, f).

Epigastric abdominal wall hernia became evident in 1 patient who had been followed at our outpatient clinic for 3 years after undergoing an emergency operation for a ruptured abdominal aortic aneurysm.

The VHPQ was answered by nine patients. Selected questions from the VHPQ are shown in Table 3. Seven out of nine patients were satisfied with this procedure, and none complained of pain, or were limited in their daily activities.

**Table 3:** Selected questions from VHPQ

Pain right now—not easily ignored	0/9
Pain last week—not easily ignored	0/9
Abdominal stiffness/rigidity	0/9
Satisfaction of the operation	7/9

*VHPQ* ventral hernia pain questionnaire

## Discussion

We previously demonstrated the safety and effectiveness of early abdominal reconstruction using turnover flap method [6, 9, 10]. In the present study, this procedure was also shown to be safe and effective based on the results of the long-term follow-up.

OA is reported to improve the prognosis of critically ill patients [1]. However, it has been reported that it can be difficult to achieve primary fascial closure after more than 7 days in OA patients because of the lateralization of the abdominal musculatures [2] and that prolonged duration of OA may result in severe complications, including the development of an abscess, enteric fistula, graft infection, and extensive ventral hernia [1, 2]. Thus, the clinical practice guidelines [3, 4] recently recommend that abdomen should be definitively closed as soon as possible. If early definitive closure cannot be achieved in a patient with OA, planned ventral hernia will be selected. In this case, it takes 6 to 12 months to perform late abdominal wall reconstruction, and it may represent a challenge for procedure to resolve the adhesion and reconstruction [11]. Moreover, although various techniques have been used in the final phase of abdominal wall reconstruction, the recurrence rates are reported to be up to 54% [12]. Thus, early abdominal wall reconstruction may be proposed to avoid severe complications in acute phase of OA.

Ventral hernia is associated with discomfort. We experienced one case of the ventral hernia, in a patient who had been followed in our outpatient clinic for 3 years after undergoing an emergency operation for a ruptured abdominal aortic aneurysm. In the questionnaire of the VHPQ, the patient was unsatisfied with this procedure, but he cited no pain or limitations in his daily activities.

The turnover flap method was performed by separating the skin and subcutaneous tissue from the anterior rectus sheath; however, we did not experience any cases of severe wound complications. Although one patient developed a major wound infection, the approximated fascial flap appeared to be intact. Turnover flaps are only a thin membrane of fascia; however, we do not need to use the prosthetic material to reinforce the turnover flaps. This may be advantageous, especially in OA patients with abdominal wall or cavity contamination due to abdominal sepsis, or an extended period of OA.

In the present study, the turnover flap method was performed to treat 2 trauma and 13 non-trauma patients. Among the trauma patients, although all bowel injuries were repaired by a deferred anastomosis, no contamination of the abdominal wall or cavity was recognized. Consequently, neither complications nor complaints regarding this procedure were seen after management with the turnover flap method. Although the etiologies of OA in non-trauma patients varied, three patients suffering from diffuse peritonitis were recognized to have intra-abdominal contamination despite successful source control: two due to perforation of colonic disease and one due to urinary bladder injuries and purulent urine in the abdomen. As a result, a major wound infection was observed in one patient; however, all patients survived hospitalization, and no other complications were seen. Two of these three contaminated patients were able to be evaluated for their long-term outcome and were satisfied with this procedure. Thus, the turnover flap method was suggested to be applicable in trauma and non-trauma patients. However, further studies will be needed to confirm these findings.

Although the anatomical features varied widely among patients, it should be kept in mind that there is not the posterior rectus sheath below the arcuate line [13] in the lower abdomen. The largest fascial gap is in the mid-abdomen, and a wide flap is needed to approximate the fascia in most patients. In contrast, although there is no posterior rectus sheath that is below the arcuate line, the fascial gap is not as wide as that of the mid-abdomen. Therefore, we create the turnover flap from the lateral border of the anterior rectus sheath to the site approximately 2 cm outside linea alba based on the width of fascial gap, and then the area of the unseparated fascia becomes a hinge in the lower abdomen. Up to now, no problems have been experienced when performing this procedure.

In the present study, the turnover flap method was performed in order to achieve early abdominal closure and avoid severe complications. CS technique, which has mostly been used in elective abdominal wall reconstruction for the repair of ventral hernias, was recently reported to show favorable results when used for a similar purpose (wound infection, 3/16; fistula, 3/16; ventral hernia, 2/8) [5]. The present results suggest that the turnover flap method was not inferior to CS technique.

### Limitations

The present study is associated with several limitations. We analyzed a small series over a long period of time (13 years), during which many things changed concerning the management of critically ill patients. Its retrospective, single-center design means that there was no strict algorithm protocol for the management of OA (e.g., the choice of TAC or the timing of turnover flap method) and that the clinical follow-up data were insufficient. Relatively few cases were evaluated with the VHPQ.

## Conclusion

In the present study, we evaluated the long-term outcomes of early abdominal wall reconstruction using the turnover flap method. Although 3 out of 15 patients developed wound infection, no other complications due to a prolonged duration of OA were detected during hospitalization. One patient was confirmed to have developed a ventral hernia in physical examinations and on CT. Based on the results of the VHPQ, seven out of nine patients were satisfied with this procedure, and none complained of pain or were in their limited daily activities. These observations suggested that early abdominal reconstruction using the turnover flap method might be considered to be safe and effective as an alternative technique that can be applied in the treatment of OA patients in whom definitive fascial closure is difficult to achieve. However, further studies are needed. A larger, multi-center, and prospective (ideally randomized) study comparing this technique of closure of OA, with other techniques of closure (using mesh [biological/synthetic]), will provide more conclusive findings.

## Abbreviations

CS: Component separation
MOF: Multiple organ failure
NPWT: Negative pressure wound therapy
OA: Open abdomen
TAC: Temporary abdominal closure
turnover flap method: Bilateral anterior rectus abdominis sheath turnover flap method
VHPQ: Ventral hernia pain questionnaire
